# High-Throughput Micro-Combinatorial TEM Phase Mapping of the DC Magnetron Sputtered Y_x_Ti_1−x_O_y_ Thin Layer System

**DOI:** 10.3390/nano14110925

**Published:** 2024-05-24

**Authors:** Dániel Olasz, Viktória Kis, Ildikó Cora, Miklós Németh, György Sáfrán

**Affiliations:** 1 Institute for Technical Physics and Materials Science, HUN-REN Centre for Energy Research, Konkoly-Thege Miklós út 29-33, 1121 Budapest, Hungary; olasz.daniel@ek.hun-ren.hu (D.O.); kis.viktoria@ek.hun-ren.hu (V.K.); ildiko.cora@ek.hun-ren.hu (I.C.); 2 Department of Materials Physics, Eötvös Loránd University, 1518 Budapest, Hungary; 3 Department of Mineralogy, Eötvös Loránd University, Pázmány Péter sétány 1/c, 1117 Budapest, Hungary; 4 Surface Chemistry and Catalysis Department, HUN-REN Centre for Energy Research, Hungarian Academy of Sciences, Konkoly-Thege Miklós út 29-33, 1121 Budapest, Hungary; nemeth.miklos@ek.hun-ren.hu

**Keywords:** Y-Ti-O thin films, micro-combinatory, pyrochlore structure, TEM, phase map, reactive DC sputtering

## Abstract

High-throughput methods are extremely important in today’s materials science, especially in the case of thin film characterization. The micro-combinatorial method enables the deposition and characterization of entire multicomponent thin film systems within a single sample. In this paper, we report the application of this method for the comprehensive TEM characterization of the Y-Ti-O layer system. Variable composition samples (Y_x_Ti_1−x_O_y_) were prepared by dual DC magnetron sputtering, covering the entire (0 ≤ x ≤ 1) concentration range. The structure and morphology of phases formed in both as-deposited and annealed samples at 600, 700, and 800 °C were revealed as a function of Y-Ti composition (x). A comprehensive map showing the appropriate amorphous and crystalline phases, and their occurrence regions of the whole Y-Ti-O layer system, was revealed. Thanks to the applied method, it was shown with ease that at the given experimental conditions, the Y_2_Ti_2_O_7_ phase with a pyrochlore structure forms already at 700 °C without the TiO_2_ and Y_2_O_3_ by-phases, which is remarkably lower than the required temperature for most physical preparation methods, demonstrating the importance and benefits of creating phase maps in materials science and technology.

## 1. Introduction

The properties of multicomponent materials, whether they are bulk substances or thin films, are essentially determined by the properties and proportion of constituent elements. Contrary to the classical, low-efficiency, one experiment–one composition philosophy, combinatorial approaches provide the synthesis of a series of compounds within a single manufacturing process and allow the characterization of multicomponent thin layer systems very efficiently by a series of analytical methods, such as X-ray diffraction, scanning electron microscopy, energy dispersive spectroscopy (EDS), spectroscopic ellipsometry, nanoindentation, etc. [1,2,3,4]. Unfortunately, most combinatorial techniques do not directly allow transmission electron microscopy (TEM), which is one of the most suitable tools for microstructural analysis as the examination of each composition of the obtained samples requires additional TEM sample preparation. With the novel micro-combinatorial method [5,6], however, a variable layer over the entire composition range can be deposited on a single TEM grid, which allows high-throughput comprehensive TEM characterization without the need for additional sample preparation.

Our method has so far been used to study the morphology, structure and material properties of a number of multicomponent films with variable compositions, e.g., Si-Ge, Hf-O-N, Al-Cu, Si-O-N, etc. [7,8,9,10].

In this study, exploiting the efficiency of the micro-combinatorial approach, we revealed for the first time in the literature a complete phase map of a multicomponent thin layer system valid for the whole composition, and over a wide temperature range up to 800 °C.

The Y-Ti-O ternary thin layer system has been widely studied due to its microstructure, material properties and variety of applications. From a technological point of view, two specific crystal structures of yttrium titanate are worth mentioning—the perovskite and the pyrochlore structure. Materials with a perovskite structure have received much attention in semiconductor research recently [11]. Their chemical formula is ABX_3_, where “A” and “B” are cations and X is an anion that binds to both. Many elements, including yttrium and titanium, can be combined to form perovskite structures that exhibit a wide range of physical, optical and electrical properties. Perovskite solar cells can be manufactured by simple, additive deposition techniques like printing for a fraction of the cost and energy compared to traditional silicon technology [12]. Recently, the photo-conversion efficiency of perovskite solar cells has exceeded 25%, close to that of silicon (27%). In addition, their magnetoresistance at room temperature has also received much interest [13,14,15].

Yttrium titanate (Y_2_Ti_2_O_7_) with a pyrochlore structure has attracted increasing interest in recent years [16,17,18,19]. Pyrochlores (s.g. *Fd*$\bar{3}$*m*) are fluorite structure-based binary oxides having a general formula of A_2_B_2_O_7_, where A is a rare earth metal ion with a coordination number of 8 and B is a transition metal with a coordination number of 6. 

Y_2_Ti_2_O_7_ exhibits excellent mechanical and chemical properties, as well as thermal stability, making it a promising candidate for various technological applications. TiO_2_ and Y_2_O_3_, for example, have traditionally been applied in oxide-dispersed strengthened (ODS) alloys; however, in the last few years there have been a number of publications on the beneficial use of Y_2_Ti_2_O_7_ in ODS materials. This is because, in addition to increased hardness, high resistance to radiation damage makes these materials suitable for nuclear reactor applications [20,21,22,23,24]. It also shows potential in nuclear waste management as it immobilizes actinides [25,26], and has remarkable applications in solid fuel cells [27] as a dielectric material [28] or as a photocatalyst [29]. 

Most of the techniques for the synthesis of the Y_2_Ti_2_O_7_ phase can be divided into two main groups: (i) mechanically assisted annealing methods and (ii) wet chemical methods. For the former one, for example, milling and hot isostatic pressure techniques are used [30,31] for TiO_2_ and Y_2_O_3_ powders in a 2:1 molar ratio, where a typically long (~few hours) and high-temperature (>1000 °C) annealing process takes place. Wet chemical methods, such as the co-precipitation technique and sol–gel method, usually allow lower synthesis temperature (~800 °C) alternatives [32,33]; however, they can be more time-consuming and demanding, complicated methods. At the temperatures required for the formation of yttrium titanate, by-phases (typically TiO_2_ and Y_2_O_3_) may still be present and disappear only at higher synthesis temperatures. For example, a low-temperature route was presented by Wang et al. [34], who prepared yttrium titanate nanoparticles upon annealing Y-Ti hydride. At 700 °C, the X-ray diffraction pattern showed weak peaks characteristic of yttrium titanate, but only annealing at 900 °C resulted in a pure yttrium titanate phase, free from TiO_2_ and Y_2_O_3_. Karthick et al. [33] synthesized single-phase yttrium titanate by annealing a ball-milled TiO_2_-Y_2_O_3_ powder mixture at 900 °C and at a fast 10 min/700 °C heat treatment using the reverse co-precipitation technique.

In this work, the novel micro-combinatorial approach was applied for the characterization of the yttrium titanate thin layer system. Another novelty is the applied synthesis method for the Y-Ti-O system, the reactive DC magnetron sputtering. Our aim was to reveal the correlations between composition, structure and annealing temperature and thus to create a phase map that covers the entire Y-Ti-O thin film system regarding the Y/Ti ratio. The micro-combinatorial technique enabled a comprehensive study via a high-throughput synthesis and TEM characterization of a variable composition of Y_x_Ti_1−x_O_y_ layers covering the whole concentration range (0 ≤ x ≤ 1) within a single TEM grid.

## 2. Materials and Methods

### 2.1. Sample Preparation

Composition-spread Y-Ti-O samples were prepared via reactive dual DC magnetron sputtering of Y and Ti targets in a stainless steel ultra-high vacuum system. The base pressure of the chamber was $3\times 10^{-8}\;mbar$, and the partial pressures of the working and reactive gases were $3\times 10^{-3}\;mbar$ Ar and $1\times 10^{-4}\;mbar$ $O_{2}$, respectively. To deposit a gradient Y-Ti oxide layer, the power of the Y and Ti sources was controlled between 0 and 275 W and between 200 and 0 W, respectively, in sync with the movement of a shutter with a narrow (100 μm) slot, over the 3 mm diameter substrate suitable for TEM. During sputtering, the substrate holder was on ground potential. As a result, a thin, variable composition Y_x_Ti_1−x_O_y_ layer (0 ≤ x ≤ 1) with dimensions of 2 × 1 mm^2^ was achieved. In a single vacuum run, multiple (4 pcs) combinatorial TEM samples of ~35 nm thickness were prepared simultaneously on Au TEM grids coated with a SiON support layer. The set of 4 pcs of composition spread samples enabled a comprehensive TEM investigation of the entire Y-Ti-O thin layer system in both deposited and annealed states, in a wide temperature range between room temperature and 800 °C.

**Figure 1:**
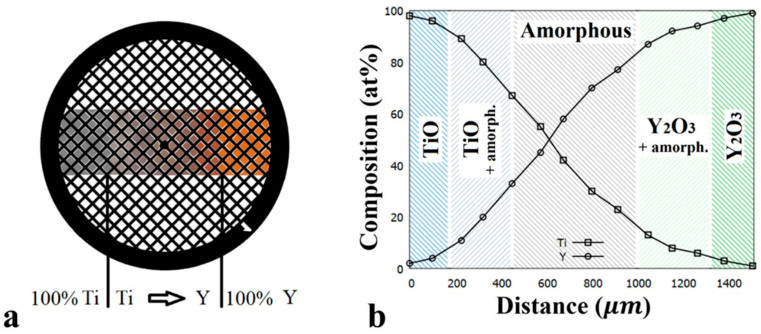
(**a**) Schematic of the as-deposited micro-combinatorial Y-Ti-O sample on TEM grid; (**b**) concentration profile of Y and Ti measured by TEM EDS along the variable composition strip.

A schematic image of the sputter-deposited micro-combinatorial sample and the concentration profile of Y and Ti, measured by TEM EDS (energy dispersive spectrometry) along the strip of variable composition, is shown in Figure 1a,b.

Sample 1 was characterized as deposited, while the other 3 samples were first subjected to heat treatments at various temperatures. Preliminary heating experiments below 600 °C showed no remarkable microstructural changes in the Y-Ti-O layers, so the minimum annealing temperature was set at 600 °C. Samples 2, 3 and 4 were heat-treated at 600, 700 and 800 °C, respectively. The samples were mounted on a stainless-steel TEM-grid holder and annealed in a Micromeritics Autochem instrument’s quartz reactor in a gas mixture of He and 10% O_2_ (both 5.0 purity). The heating rate was 10 °C/min and after the sample reached the selected temperature, it was annealed for 1 h.

### 2.2. Characterization Methods

The characterization of the composition spread samples deposited on microgrids was carried out by TEM using a Thermo Fisher (Waltham, MA, USA) Titan Themis 200 kV spherical aberration (Cs)-corrected TEM/STEM microscope having 0.08 nm high-resolution TEM and 0.16 nm scanning TEM point resolution, equipped with 4 Super-X EDS detectors. Structure and morphology of the various phases as a function of compositions were revealed by selected area electron diffraction (SAED) and bright field (BF) imaging along with EDS measurements. SAED measurements were carried out following the procedure of Czigány and Kis [35], which allows high (0.1%) accuracy without using an internal calibration sample. SAED patterns were evaluated with the ProcessDiffraction software (v 12.11.3) [36]. TEM characterization of the sample was performed at representative sites selected along the Ti-Y concentration gradient for as-deposited as well as the ex situ annealed samples. The occurrence ranges of the various Y-Ti-O phases as a function of composition and annealing temperature were compiled into a phase map.

It should be noted that in the presence of crystalline rings in electron diffraction, it is challenging to detect and analyze faint, diffuse rings of small amounts of amorphous phase. For this reason, the boundaries between crystalline and amorphous phases in the map were defined according to the appearance or disappearance of the crystalline phase of interest.

## 3. Results

### 3.1. As Deposited Y-Ti-O Sample

TEM investigation of the as-deposited combinatorial sample No. 1, carried out at carefully selected sites along the variable composition layer, revealed the morphology and structure of the various Y-Ti-O phases formed as a function of the Y:Ti ratio. Based on detailed TEM and EDS analyses at a large number of measurement sites, it was possible to identify all the phases that form in the Y-Ti-O layer system as a function of local composition under the given conditions, and to determine their concentration range of occurrence.

**Figure 2:**
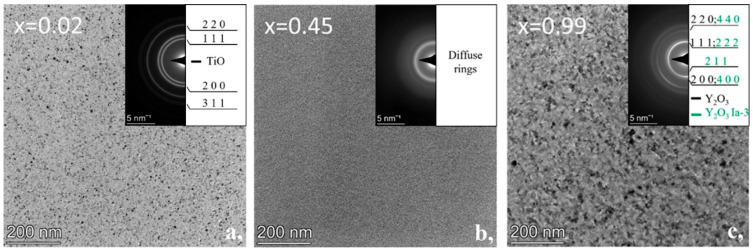
As-deposited Y_x_Ti_1−x_O_y_ sample. BF TEM images of the sample at (**a**) x = 0.02, (**b**) x = 0.45, (**c**) x = 0.99 compositions. Insets are corresponding SAED patterns representing (**a**) polycrystalline TiO, (**b**) amorphous YTiO, (**c**) polycrystalline Y_2_O_3_ phases.

**Figure 3:**
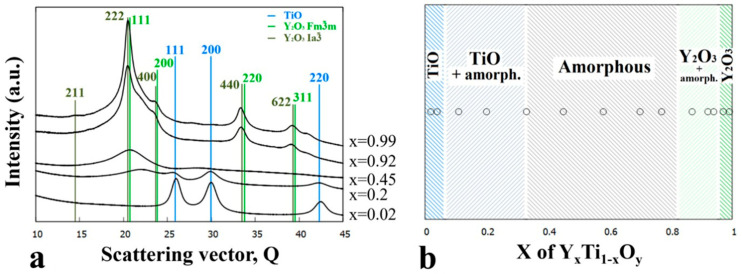
(**a**) Integrated 1D SAED intensity profiles for indicated (x) compositions and characteristic peaks of the TiO (*Fm*$\bar{3}$*m*), Y_2_O_3_ (*Fm*$\bar{3}$*m*) and Y_2_O_3_ (Ia$\bar{3}$) phases and (**b**) phase map of the as-deposited Y-Ti-O sample as a function of Y concentration (x).

Figure 2 shows typical bright field images and corresponding SAED patterns of the three single-phase regions of the as-deposited sample, while Figure 3 is a compilation of both. At the Ti surplus side, a fine crystalline TiO phase with grain sizes up to ~10 nm is present up to about 6~7 at% Y. It exhibits a cubic NaCl (rock salt) type structure (s.g. *Fm*$\bar{3}$*m*, a=4.197 Å), as shown by the BF TEM micrograph with SAED inset in Figure 2a. The TiO phase is present at increasing Y concentrations up to x = 0.33 (33 at% Y); however, it is associated with an amorphous phase entering at about 6~7 at% Y. At 33 at% Y, already, the amorphous phase dominates, showing negligible signs of the presence of TiO crystallites. The exclusively amorphous region is represented by Figure 2b, taken at 45 at% Y, which shows typical salt-and-pepper type contrast and diffuse SAED rings (amorphous peak maximum at 2.91 Å (Q = 21.6 nm^−1^)). The amorphous region extends over a wide range up to a Y concentration of 87 at% (x = 0.87), where the crystalline rings of the CaF_2_-type cubic structure phase Y_2_O_3_, (s.g. *Fm*$\bar{3}$*m*, a=5.264 Å) appear. The Y_2_O_3_ phase was found to be present up to 100 at% Y concentration, so that up to 97 at% an amorphous by-phase was present. Above 97 at% Y, the crystalline Y_2_O_3_ up to ~20–30 nm grain sizes was found with no additional amorphous phase, as represented by Figure 2c. It is worthwhile to note that at a small amount of Ti (~1 at%), SAED also shows a distinct ring (Figure 2c {211} ring—marked with green) of the Y_2_O_3_, Mn_2_O_3_-type, Ia$\bar{3}$ phase. This phase has also a CaF_2_-based structure, but with a lattice parameter twice as large (*a* = 10.598 Å) as that of the CaF_2_- type Y_2_O_3_ phase.

Figure 3a shows a series of integrated 1D intensity profiles of SAED patterns for compositions x = 0.02, 0.2, 0.45, 0.92, and 0.99, selected to represent the revealed phase regions. The marked characteristic peaks were used for phase identification. In Figure 3b, the phase map compiled from the TEM results shows the Y-Ti-O phases formed and their ranges of occurrence in the as-deposited sample as a function of Y concentration (x).

### 3.2. Sample Annealed at 600 °C

**Figure 4:**
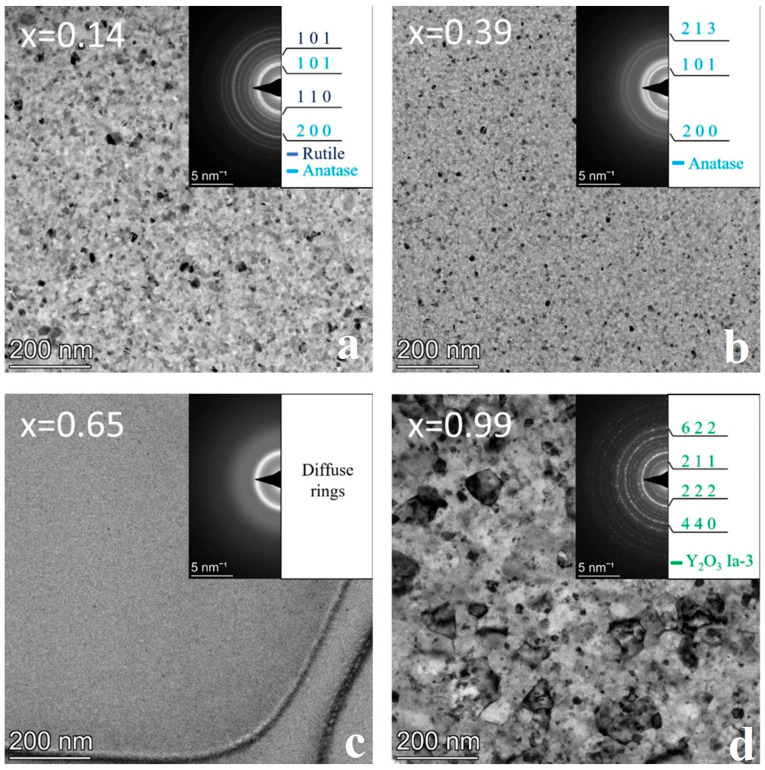
BF TEM images with SAED insets of the sample annealed at 600 °C and at compositions (**a**) x = 0.14, (**b**) x = 0.39, (**c**) x = 0.65, and (**d**) x = 0.99 showing the characteristic microstructure for different phase regions.

**Figure 5:**
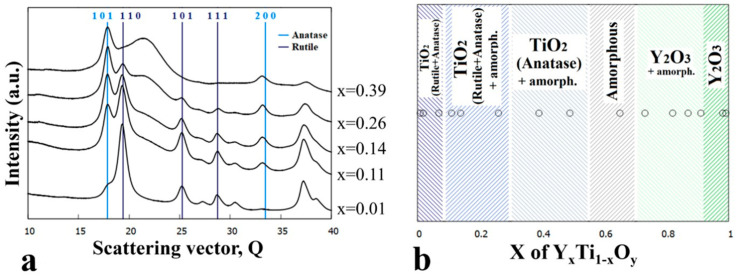
(**a**) 1D intensity profiles showing the different intensity ratios between peaks of the rutile and anatase phases as the yttrium concentration increases (x = 0.01–0.39) and (**b**) phase regions as a function of composition x for the sample annealed at 600 °C.

The as-deposited combinatorial sample No. 2 was subjected to annealing at 600 °C for 1 h in He-O_2_ gas mixture followed by TEM characterization. The microstructures of the different phase regions of the composition spread sample are represented by bright field images and corresponding SAED patterns for Y concentrations x = 0.14, 0.39, 0.65, and 0.99 (Figure 4). The resulting phase map is shown in Figure 5b.

The 600 °C heat treatment in the O-containing He atmosphere results in further oxidation of TiO to TiO_2_, which, depending on the composition, appears as both rutile (P4_2_/mnm space group, *a* = 4.59 Å, c = 2.96 Å) and anatase (I4_1_/amd, *a* = 3.73 Å, c = 9.37 Å). In addition, a remarkable increase in the grain size was found with respect to the as-deposited sample, up to ~30 nm and ~100 nm, as shown in Figure 4a,d, respectively. After heat treatment, the previously amorphous film at x = 0.39 Y concentration becomes nanocrystalline (~10 nm in size); however, up to around x = 0.6 the amorphous structure is preserved (Figure 4c).

The structural and morphological changes are also clearly recognized by comparing the diffracted intensity profiles in Figure 3a and Figure 5a. The ratio of rutile and anatase phases is influenced by the Y concentration in the x ≅ 0–0.4 composition range, as illustrated by Figure 5a. At low Y concentrations, the rutile phase dominates, whereas with increasing Y concentration, the intensity of the peaks of anatase increases. At x = 0.26, the peaks of the anatase overwhelm that of rutile, and at x = 0.39, only peaks for the anatase phase are present. In parallel to the increase of the anatase volume ratio, the gradual increase of an amorphous peak, with its maximum positioned at 2.93 Å (Q = 21.4 nm^−1^), can be observed. The heat treatment at 600 °C resulted in a significant reduction in the width of the single-phase amorphous region mainly in favor of TiO_2_ anatase, and to a smaller extent of Y_2_O_3_. Compared to the as-deposited state shown in Figure 3, the {211} ring of the Y_2_O_3_ phase with the Ia$\bar{3}$ space group became more intense (see Figure 4d), indicating an increased volume fraction.

### 3.3. Sample Annealed at 700 °C

**Figure 6:**
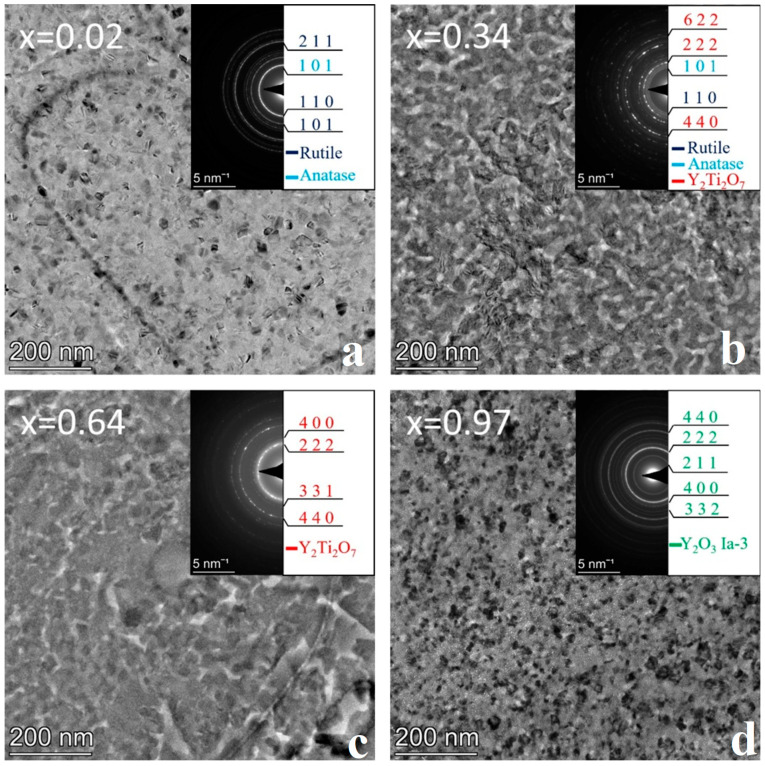
TEM images and SAED insets of the Y_x_Ti_1−x_O_y_ sample annealed at 700 °C at compositions (**a**) x = 0.02, (**b**) x = 0.34, (**c**) x = 0.64, and (**d**) x = 0.97 showing the characteristic microstructures for the different phase regions.

The as-deposited combinatorial sample No. 3 was subjected to annealing at 700 °C for 1 h prior to TEM characterization. Figure 6a–d show representative TEM images and SAED insets taken at different Y:Ti ratios (x = 0.02, 0.34, 0.64, 0.97). The typical grain sizes for the different compositions are about 30 nm, 50 nm, 120 nm, and 40 nm, respectively. An exclusively amorphous region covering a narrow concentration range (~x = 0.66–0.74) was still observed (not shown here by TEM image).

**Figure 7:**
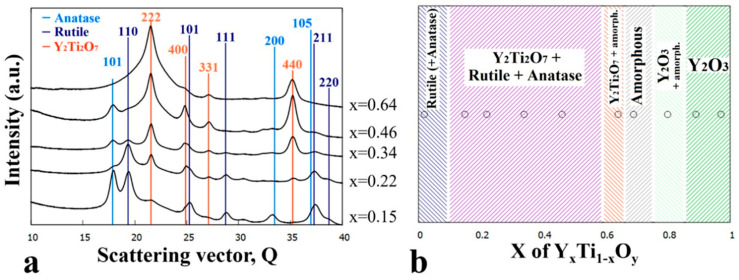
(**a**) 1D intensity profiles of SAED images for the x = 0.15–0.64 composition range and (**b**) phase regions as a function of composition (x) for the Y_x_Ti_1−x_O_y_ sample annealed at 700 °C.

Figure 7b depicts the corresponding phase map as a function of Y composition. At very low yttrium concentrations (~x = 0.02) the rutile phase is dominant; however, weak rings corresponding to the anatase phase (Figure 6a: {101} ring) are also recognized. By increasing the Y concentration (above ~x = 0.15), a three-phase region is found where, in addition to anatase and rutile, the pyrochlore-type yttrium titanate Y_2_Ti_2_O_7_ (s.g. *Fd*$\bar{3}$*m*, *a* = 10.09 Å) appears. A further increase of the Y concentration toward and above x = 0.5 leads to an increase in the pyrochlore-type yttrium titanate phase fraction at the expense of rutile and anatase. As represented by the SAED in Figure 6c, the Y_2_Ti_2_O_7_ phase appears as only a crystalline phase around the x = 0.64 Y concentration. The width and bluntness of the peaks in the integrated SAED intensity profiles suggest an amorphous by-phase in addition to the crystalline Y_2_Ti_2_O_7_ (yttrium titanate). Figure 7a shows the evolution of the intensity profile of SAEDs with increasing Y concentration.

It should be noted that the appearance of the crystalline Y_2_Ti_2_O_7_ occurs at the expense of the amorphous phase; therefore, the concentration range of the amorphous region is reduced (~x = 0.66–0.74), as shown in Figure 7b. The upper concentration border between the amorphous and Y_2_O_3_ (Ia$\bar{3}$) + amorphous by-phase region did not shift significantly. In order to overview the development of phases upon annealing at 700 °C, selected integrated SAED 1D intensity profiles are plotted in the relevant concentration range of x = 0.15 to x = 0.64 in Figure 7a.

### 3.4. Sample Annealed at 800 °C

**Figure 8:**
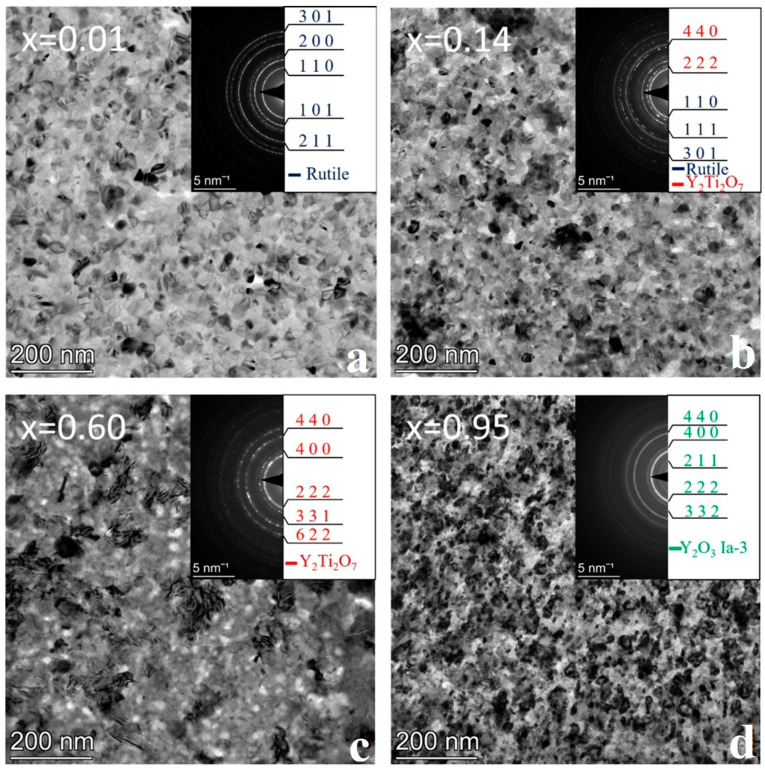
BF TEM images and corresponding SAED insets of the sample annealed at 800 °C and at compositions (**a**) x = 0.01, (**b**) x = 0.14, (**c**) x = 0.60, and (**d**) x = 0.95 showing the characteristic microstructure for the different phase regions.

Annealing of sample No. 4 at 800 °C for 1 h resulted in complete crystallization of the entire Y-Ti-O layer system. Figure 8 shows a selection of bright field TEM images with SAED pattern insets at different compositions representative of the phases formed. The bright field images in Figure 8a,d show that the grain size increases to ~40 nm and ~40–60 nm at the ends of the sample containing TiO_2_ and Y_2_O_3_, respectively. This shows no significant increase in grain size compared to sample No. 3 heat-treated at 700 °C.

However, a comparison of the SAED inserts in Figure 6c and Figure 8c shows that the rings corresponding to the yttrium titanate phase of Y_2_Ti_2_O_7_ became much more prominent due to the 800 °C annealing, indicating a higher degree of crystallization.

**Figure 9:**
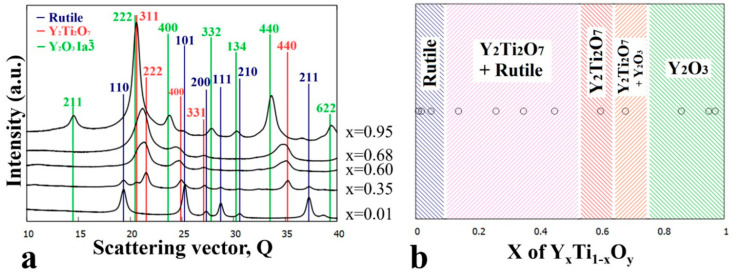
(**a**) 1D intensity profiles of SAED images for the composition range x = 0.01–0.95 and (**b**) arrangement of phases along the micro-combinatorial TEM sample (as a function of composition x) annealed at 800 °C for 1 h.

The phase map of the whole composition range of the 800 °C annealed sample is shown in Figure 9b. At the Ti-rich side, up to ~x = 0.08 exclusively rutile phase of TiO_2_ is present. Next to that, as the Y concentration increases, rutile and yttrium titanate (Y_2_Ti_2_O_7_) are simultaneously present (x = ~0.08–0.52). This is to note that regardless of the composition, the anatase phase of TiO_2_ disappeared completely from the sample due to the 800 °C heat treatment. A single-phase crystalline yttrium titanate with a pyrochlore structure (Y_2_Ti_2_O_7_) is present and can be clearly observed in the SAED image (Figure 8c) at the x = 0.60 composition. It is of interest that, contrary to an increase in grain size, the individual rings in the SAED became slightly diffuse. This could be explained by a stress built into the film causing local crystal deformation. It is reflected in the frequent contrast changes within the grains, as recognized in BF TEM in Figure 8c. The stress incorporation may be due to a deviation from the stoichiometry, as the composition in the study area (Figure 8c) is x = 0.60 instead of x = 0.50, typical for the Y_2_Ti_2_O_7_ phase.

Note that the sample annealed at 800 °C no longer contains an amorphous phase. The comparison of Figure 7b and Figure 9b shows that at about the previously amorphous region, a mixture of crystalline Y_2_Ti_2_O_7_ and Y_2_O_3_ phases is present.

## 4. Discussion and Conclusions

The morphology and structure of the as-deposited and heat-treated combinatorial samples were revealed by TEM and SAED as a function of Y-Ti composition and annealing temperature. The phases were identified and the results are shown in the partial phase maps in Figure 2, Figure 3, Figure 4, Figure 5, Figure 6, Figure 7, Figure 8 and Figure 9. 

**Figure 10:**
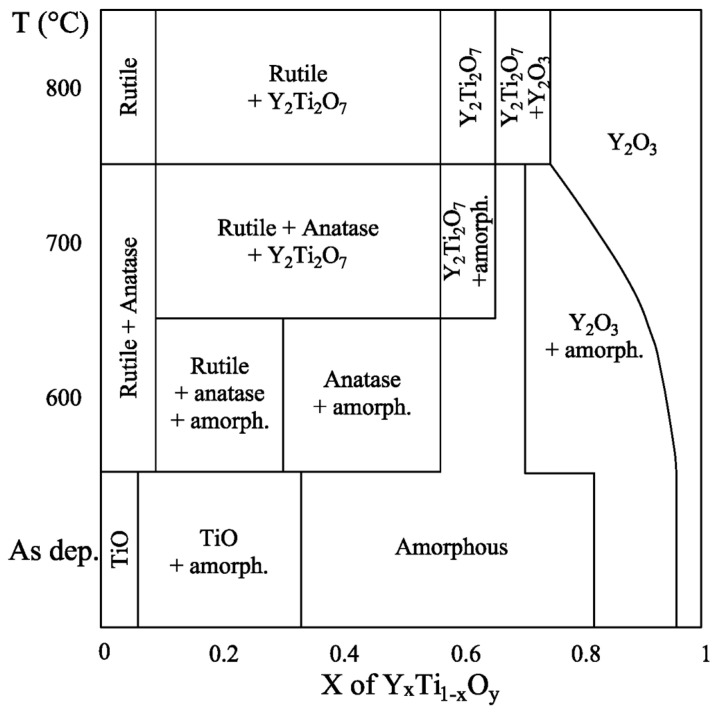
Compiled phase map of the reactive DC magnetron sputtered Y_x_Ti_1−x_O_y_ thin film system over the entire Y/Ti composition range, up to 800 °C.

By merging these sub-maps, we created a complete phase map of the Y-Ti-O layer system up to 800 °C. This is the main result of our paper, illustrated in Figure 10. 

The phase map reveals information on the relevant phases, their positions and the Y concentration (x) range they cover as a function of annealing temperature. 

A brief overview of the phase map is given below. Both the Y and Ti surplus sides of the map with low foreign components concentration show crystalline phases regardless of temperature. At the Ti-rich side, the TiO, rutile + anatase, and rutile phases are present at room temperature (RT) (in as-deposited state), at 600 and 700 °C, and at 800 °C annealing temperatures, respectively. These phases incorporate about 5~8 at% Y (x = 0.05…0.08). At the Y-rich side of the map, with increasing temperature, the width of the crystalline Y_2_O_3_ region regarding x increases from 0.95…0.75. Between these two side regions, an amorphous phase is present alone or with crystalline phases up to 700 °C, which covers smaller and smaller areas as the temperature increases, suggesting that crystalline phases are favored with elevated temperatures. Above 700 °C exclusively, crystalline phases show up. 

At room temperature, the (RT) TiO + amorphous phases are present between x = 0.05 and 0.32 Y concentrations. In the middle range (x = 0.32…0.82), only the amorphous phase is found, while between x = 0.82 and x = 0.95, the Y_2_O_3_ + amorphous phases are present. 

Layers annealed at 600 °C exhibit six phase regions: rutile + anatase in the range of x = 0…0.08, rutile + anatase + amorphous at x = 0.08…0.3, anatase + amorphous at x = 0.3…0.55, only amorphous phase at x = 0.55…0.7, Y_2_O_3_ + amorphous at x = 0.7…0.95, and Y_2_O_3_ at 0.95…1. 

At 700 °C, at the Ti side of the map, the rutile + anatase phases are present up to 8 at% Y concentration (x = 0…0.08). Above this Y concentration, the pyrochlore yttrium titanate Y_2_Ti_2_O_7_ phase appears in addition to rutile and anatase and is present up to a Y concentration of around 55 at% (x = 0.08…0.55). Note that 700 °C is a surprisingly low temperature [33,34] for the formation of the pyrochlore phase. With increasing Y concentrations (x = 0.55…0.66), a two-phase region appears in which the pyrochlore and amorphous phases coexist. The crystalline phases develop at the expense of the amorphous phase. The width of the pure amorphous range at this (700 °C) temperature is rather narrow, limited to Y concentrations between 66 at% and 70 at% (x = 0.66…0.7). Above this range, the crystalline Y_2_O_3_ and amorphous phases coexist up to about x = 0.8 Y concentration. From x = 0.8 up to x = 1, only crystalline Y_2_O_3_ is present. 

In the sample heated at 800 °C, the pyrochlore phase appears for the first time completely alone, without any crystalline or amorphous side-phases, in the range of 56…66 at% Y concentration. To the left are the rutile + pyrochlore and rutile phases, respectively, separated by the 8 at% yttrium concentration line. To the right of the Y_2_Ti_2_O_7_ (pyrochlore) phase range, the map shows a Y_2_Ti_2_O_7_ + Y_2_O_3_ mixed phase, followed by a pure Y_2_O_3_ phase above 74 at% Y (x = 0.74).

The shift in the appearance of the single Y_2_Ti_2_O_7_ phase toward Y excess compositions (x > 0.5) is in a very good agreement with previously reported results. Higashi et al. [29] observed that in using the polymer complex method, no TiO_2_ by-phase developed next to the pyrochlore phase in samples with 5 at% Y excess, thus increasing the photocatalytic activity of the final sample. 

For the preparation of the Y-Ti-O layer system, we applied reactive magnetron sputtering. Because sputtered thin films, unlike bulk materials, are not equilibrium systems, they may behave differently to form non-equilibrium phases, or relevant phases may appear at different compositions and temperatures or even be absent. Under the given experimental conditions, it was found that the pure pyrochlore structure Y_2_Ti_2_O_7_, a stable, equilibrium yttrium titanate phase, free from by-phases such as TiO_2_ and Y_2_O_3_, could form at as low as 700 °C instead of the typical 900 °C [33,34].

It should be noted that at the current reactive DC magnetron sputter deposition experiments, the partial pressure of oxygen (the reactive component) was limited to 3 × 10^−4^ mbar. This is the maximum O_2_ partial pressure above which the targets become poisoned and sputtering effectively stops. Subsequent annealing conditions were selected to avoid oxygen deficit and favor stable structures of the yttrium titanate films. Therefore, the annealing was carried out in an O-containing He atmosphere. These conditions are believed to be the reason why one possible phase, the metastable, low-oxygen content yttrium-titanium-oxide with a perovskite structure (YTiO_3_), was not formed.

In summary, by using the high-throughput micro-combinatorial technique, the complete Y_x_Ti_1−x_O_y_ layer system could be efficiently prepared and TEM-characterized within a single sample. This allowed for the first time in the literature the compilation of a complete phase map of the DC sputter deposited Y-Ti-O layer system up to 800 °C, as shown in Figure 10. This phase map reveals the ranges of occurrence of Y-Ti-O phases in thin films and allows the appropriate parameters to be set to reproduce all the phases, which can serve as a useful reference for researchers and technologists.

Beyond Y-Ti-O, we believe that the present results highlight the potential of the micro-combinatorial approaches for the comprehensive characterization of multicomponent thin film systems, including phase mapping.

## Data Availability

Data will be made available upon reasonable request.
